# PVN Blockade of p44/42 MAPK Pathway Attenuates Salt-induced Hypertension through Modulating Neurotransmitters and Attenuating Oxidative Stress

**DOI:** 10.1038/srep43038

**Published:** 2017-02-22

**Authors:** Hong-Li Gao, Xiao-Jing Yu, Kai-Li Liu, Xiao-Lian Shi, Jie Qi, Yan-Mei Chen, Yan Zhang, Juan Bai, Qiu-Yue Yi, Zhi-Peng Feng, Wen-Sheng Chen, Wei Cui, Jin-Jun Liu, Guo-Qing Zhu, Yu-Ming Kang

**Affiliations:** 1 Department of Physiology and Pathophysiology, Xi’an Jiaotong University School of Basic Medical Sciences, Key Laboratory of Environment and Genes Related to Diseases (Xi’an Jiaotong University), Ministry of Education, Xi’an 710061, China; 2 Department of Pharmacology, Xi’an Jiaotong University School of Basic Medical Sciences, Xi’an 710061, China; 3 Department of Cardiovascular Surgery, Xijing Hospital, Fourth Military Medical University, Xi’an 710032, China; 4 Department of Endocrinology and Metabolism, First Affiliated Hospital of Xi’an Jiaotong University, Xi’an 710061, China; 5 Department of Physiology, Nanjing Medical University, Nanjing 210029, China

## Abstract

The imbalance of neurotransmitters and excessive oxidative stress responses contribute to the pathogenesis of hypertension. In this study, we determined whether blockade of p44/42 MAPK pathway in the hypothalamic paraventricular nucleus (PVN) ameliorates the development of hypertension through modulating neurotransmitters and attenuating oxidative stress. Dahl salt-sensitive (S) rats received a high-salt diet (HS, 8% NaCl) or a normal-salt diet (NS, 0.3% NaCl) for 6 weeks and were treated with bilateral PVN infusion of PD-98059 (0.025 μg/h), a p44/42 MAPK inhibitor, or vehicle via osmotic minipump. HS resulted in higher mean arterial pressure (MAP) and Fra-like (Fra-LI) activity, and plasma and PVN levels of norepinephrine (NE), tyrosine hydroxylase (TH), NOX2 and NOX4, lower PVN levels of gamma-aminobutyric acid (GABA), copper/zinc superoxide dismutase (Cu/Zn-SOD) and the 67-kDa isoform of glutamate decarboxylase (GAD67), as compared with NS group. PD-98059 infusion reduced NE, TH, NOX2 and NOX4 in the PVN, and induced Cu/Zn-SOD and GAD67 in the PVN. It suggests that PVN blockade of p44/42 MAPK attenuates hypertension through modulating neurotransmitters and attenuating oxidative stress.

Hypertension is a major risk factor for cardiovascular disease and has a significant global impact on health, which leads to increased morbidity and mortality^1^. Recent studies have established that hyperactive sympathetic nerve is strongly associated with the initiation and progression of hypertension^2^,^3^,^4^. The paraventricular nucleus (PVN) is an important central integration site for the regulation of sympathetic nerve activity and blood pressure^4^,^5^. A growing number of evidences indicate that oxidative stress in the PVN plays an important role in the pathogenesis of hypertension^4^,^6^. Several studies have shown that reactive species of oxygen (ROS)(^−^OH, 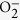, H_2_O_2_), especially 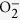 as a vital signal factor within the PVN, plays an important role in modulating blood pressure and sympathetic nerve activity. ROS are increased in the PVN of hypertensive rats and that blockade of ROS decreases sympathetic activity. NADPH oxidase is a major source of ROS in hypertension^7^,^8^ and plays a critical role in generating ROS in the brain^9^,^10^. NADPH oxidase-derived ROS is increased during the development of hypertension. Recent studies have found high salt diet produces excessive amount of ROS, such as 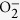 and increases the expression of superoxide protein NOX2 and NOX4 (amembrane associated oxidase protein). Blocking the production of ROS in the PVN will contribute to the prevention of hypertension.

Neurotransmitters such as glutamate, norepinephrine (NE) (a marker of sympathetic activity), and gamma-aminobutyric acid (GABA) in the PVN are known to be involved in the pathogenesis of hypertension^11^,^12^. Glutamate and NE in the PVN are the neurotransmitters that excite the sympathetic outflow^13^, and GABA in the PVN is an important neurotransmitter that inhibits sympathetic activity^14^. Evidences showed that NE and GABA in the PVN contribute to the pathogenesis of hypertension^15^,^16^,^17^, Moreover, the hypertensive responses are due to increases of excitatory adrenergic and glutamatergic activities and a decrease of GABAergic activity in the PVN^18^. There are accumulating evidences suggesting that salt-induced hypertension lead to imbalance between neurotransmitter, decreased inhibitory neurotransmitters GABA and increased excitatory adrenergic and glutamatergic in the PVN^12^,^17^. Restoring the balance between the excitatory and inhibitory neurotransmitters in the PVN is beneficial to the treatment of hypertension.

Studies over the last several decades have found that p44/42 Mitogen-activated protein kinase (MAPK), also named extracellular signal-regulated protein kinases (ERK1/2), is expressed in the PVN and associated with cardiovascular and autonomic regulation^19^,^20^. MAPK pathway is required for sympathetic activation in hypertension and is crucial for regulating cardiac physiological and pathological events^20^. The p44/42 MAPK is a major terminal effect or kinase of the MAPK family and can be activated by other factors such as proinflammatory cytokines and ROS^21^. The aim of the present study was to determine whether p44/42 MAPK in the PVN contribute to sympathetic excitation by impacting ROS levels, and restoring the balance of neurotransmitters in the PVN in HS-induced hypertensive rats. These findings will provide novel evidences for specific signaling mechanisms involved in the pathogenesis of hypertension.

## Methods

### Animals

Experiments were performed on eight-week-old male Dahl salt-sensitive (S) rats (Charles River Laboratories International, Inc., Wilmington, MA, USA). Rats were housed in a temperature-controlled room with a 12:12 light-dark cycle and allowed to access standard rat chow and tap water *ad libitum*. All protocols were approved by the Animal Care and Use Committees of Xi’an Jiaotong University and were performed in accordance with the recommendations in the Guide for the Care and Use of Laboratory Animals of the National Institutes of Health (NIH Publication No. 85–23, revised 1996).

### General experimental protocol

Rats were randomly divided into two groups receiving different diet, normal-salt diet (0.3% NaCl, NS group) and high-salt diet (8% NaCl, HS group)^22^,^23^. The bilateral PVN cannulae were implanted bilaterally as described previously^24^,^25^. One week later, the osmotic minipumps (Alzet Model 2006) were implanted subcutaneously and connected to the PVN cannulae for the continuous infusion of p44/42 MAPK inhibitor PD-98059 at 0.025 μg/h or vehicle (artificial cerebrospinal fluid, aCSF) for six weeks^26^. The success rate of the implantation operation was about 68%, and only animals with verified bilateral PVN injection sites were preceded to final analysis.

### Measurement of mean arterial pressure (MAP)

Mean arterial pressure (MAP) was measured noninvasively via tail-cuff instrument and their recording system as described previously^17^,^27^. Briefly, unanesthetized rats were warmed to an ambient temperature of 38 °C by placing rats in a holding device mounted on a thermostatically controlled warming plate. Rats were allowed to habituate to this procedure for 3 days prior to each experiment. Blood pressure was determined by a tail-cuff occlusion method, and blood pressure values were averaged from six consecutive cycles per week obtained from each rat.

### Sympathetic nerve activity (RSNA) recordings

After 6 weeks of blood pressure recorded, the rats were anaesthetized with a ketamine (80 mg/kg) and xylazine (10 mg/kg) mixture (ip) for recording RSNA. Methods for recordings and integrating RSNA have been described previously^4^,^28^.

### Collection of samples

Rats were anesthetized with a ketamine (80 mg/kg) and xylazine (10 mg/kg) mixture introperitunial (i.p.) and decapitated. Blood and tissue samples were collected. The PVN tissue was isolated following Palkovits’s microdissection procedure as previously described^17^,^29^. Samples were stored at −80 °C until assayed.

### Measurement of PVN levels of glutamate, GABA and NE, and of plasma NE

Tissue levels of NE, glutamate, GABA and plasma NE were measured using HPLC with electrochemical detection as previously described^30^,^31^,^32^.

### Immunohistochemical and immunofluorescent studies

Tissues were collected from both sides of the PVN of individual rat, sectioned into several transverse sections at about 18 μm from bregma and stored at −80 °C for next measurement. Immunohistochemical labeling was performed as previously described^27^,^32^. The primary antibodies for Fra-LI, an indicator of chronic neuronal activation, (sc-253), NOX2 (sc-20782) and NOX4 (sc-21860) were purchased from Santa Cruz Biotechnology. ROS in the PVN were determined by fluorescent-labeled dihydroethidium (DHE; Molecular Probes) staining, as previously described^3^,^17^. For each animal, the positive neurons within the bilateral borders of the PVN were manually counted similarly for data analysis in three consecutive sections and an average value was reported.

### Western blot

The PVN tissues homogenate were subjected to western blot analysis for determination of protein levels of total p44/42 MAPK (WL01864, Wanlei Biotechnology) and phosphorylated p44/42 MAPK (Thr202/Tyr204: sc-16982), NOX2 (sc-20782), NOX4 (sc-21860), antioxidant enzymes superoxide dismutase (Cu-Zn superoxide dismutase-1, SOD-1) (sc-11407), GAD67 (sc-7512) and TH (sc-14007) expression. The primary antibodies were purchased from Santa Cruz Biotechnology. The procedures of western blot were described previously^33^. Protein detection was performed using the enhanced chemiluminescence kit using Chemi Doc XRS System (Bio-Rad, USA). Protein loading was controlled by probing all Western blots with anti-β-actin antibody (Thermo Scientific, USA), and target protein intensities were normalized to that of β-actin. Band densities were analyzed using the NIH Image J software.

### Statistical analysis

Data were expressed as mean ±SEM. MAP data were analyzed by repeated-measures ANOVA. Other data were analyzed by ANOVA followed by a post-hoc Tukey test. A *P* value of less than 0.05 was considered to be statistically significant.

## Result

### Effect of PVN infusion of PD-98059, a p44/42 MAPK inhibitor on mean arterial pressure in salt-induced hypertensive rats

**Figure 1:**
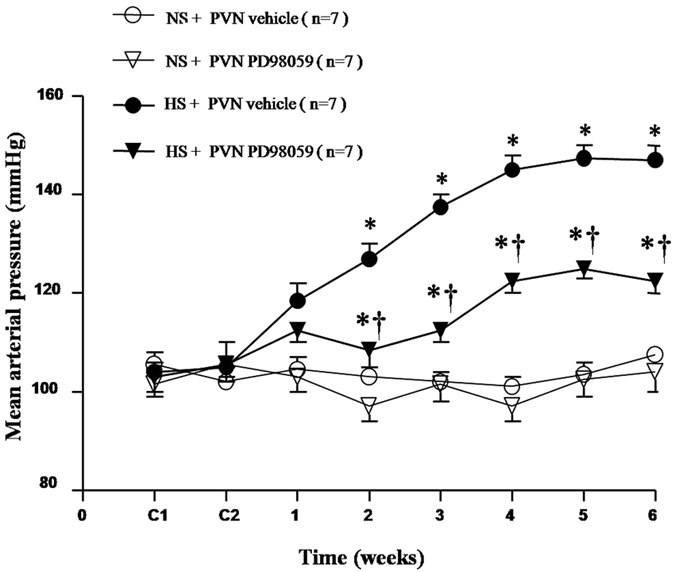
Effects of PVN infusion of PD98059 on mean arterial pressure (MAP) in salt-induced hypertensive rats. MAP in rats receiving high-salt diet increased gradually while PD98059 inhibited high salt diet-induced hypertension compared with the normal-salt (NS) diet rats (*P* < 0.05, n = 7). Values are mean ± SE. *P < 0.05 vs control (NS + PVN aCSF or NS + PVN PD98059); ^†^*P* < 0.05, HS + PVN PD98059 vs HS + PVN aCSF.

The base blood pressure before treatment among all rats was at the same level. As shown in Fig. 1, MAP increased gradually in HS rats. After two weeks of high salt intake, HS rats showed significant higher MAP compared with NS rats. With 6-week PVN infusion of PD-98059 reduced mean arterial pressure in HS rats compared with NS rats (Fig. 1, *P* < 0.05).

### Effect of PVN infusions of PD-98059 on renal sympathetic nerve activity (RSNA) and plasma norepinephrine in salt-induced hypertensive rats

**Figure 2:**
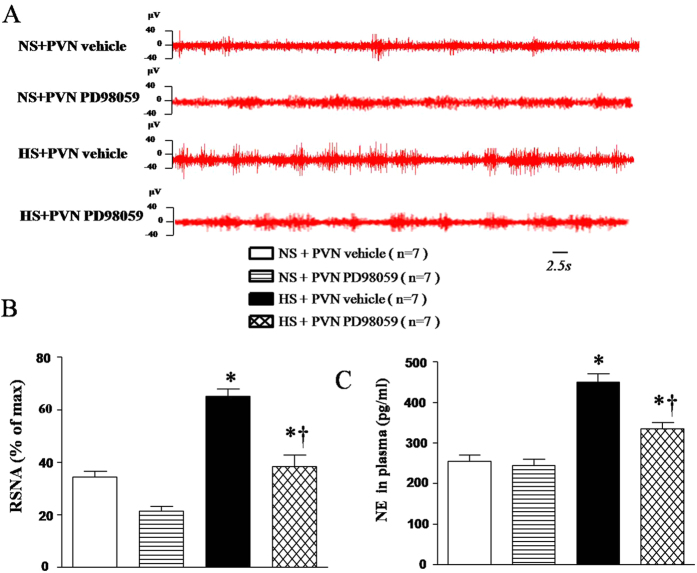
Effects of PVN infusion of PD98059 on renal sympathetic nerve activity (RSNA) and plasma norepinephrine (NE) in salt-induced hypertensive rats. High-salt diet (HS) rats exhibited a significant higher RSNA (% of max) and higher level of plasma NE compared with the normal-salt (NS) diet rats (*P* < 0.05, n = 7). When compared with HS + PVN aCSF rats, PD98059 attenuated RSNA (% of max) and plasma NE in HS rats (*P* < 0.05, n = 7). *P < 0.05 vs control (NS + PVN aCSF) or NS + PVN PD98059); ^†^*P* < 0.05, HS + PVN PD98059 vs HS + PVN aCSF.

Renal sympathetic nerve activity is an important direct indicator for the evaluation of sympathetic central activity and was measured by electrophysiological method at the end of experiment. Plasma NE is an indirect marker of sympathetic activity and was measured by HPLC. HS rats exhibited a significant higher RSNA (% of max) and higher level of plasma NE compared with NS rats. With 6-week PVN infusion of PD-98059 attenuated RSNA and plasma NE in HS rats compared with NS rats (Fig. 2, *P* < 0.05).

### Effect of PVN infusions of PD-98059 on the neuronal activity in salt-induced hypertensive rats

**Figure 3:**
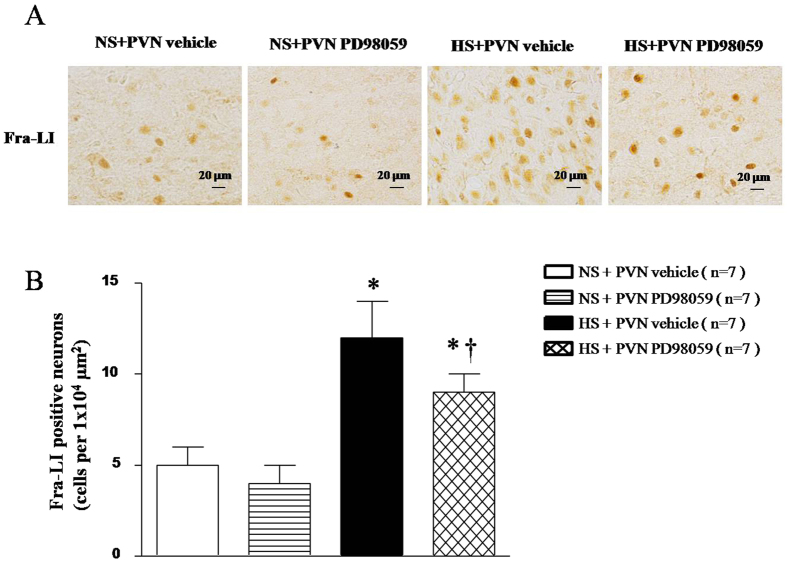
Effects of PVN infusion of PD98059 on Fra-like (Fra-LI) positive neurons within the PVN in salt-induced hypertensive rats. (**A**) A representative immunofluorescence image of Fra-LI immunoreactivity, an indicator of chronic neuronal excitation. (**B**) Densitometric analysis of Fra-LI positive neurons in PVN. PVN levels of Fra-LI immunoreactivity in High-salt diet (HS) rats were higher than in normal-salt (NS) rats (*P* < 0.05, n = 7). Chronic PVN infusions of PD98059 significantly reduced Fra-LI level in the PVN of HS rats compared with the NS rats (*P* < *0.05*, n = 7). Values are mean ± SE. *P < 0.05 vs control (NS + PVN aCSF) or NS + PVN PD98059); ^†^*P* < 0.05, HS + PVN PD98059 vs HS + PVN aCSF.

Fra-LI is an indicator of chronic neuronal activation. Immunohistochemical labeling was performed to measure Fra-LI activity in PVN. As shown in Fig. 3, HS rats had higher Fra-LI immunoreactivity in the PVN compared with NS rats. PVN treatment with PD-98059 reduced the neuronal activity in the PVN of HS rats (Fig. 3, *P* < 0.05).

### Effects of PVN infusions of PD-98059 on the oxidative stress markers and Cu/Zn-SOD protein expression in the PVN of salt-induced hypertensive rats

**Figure 4:**
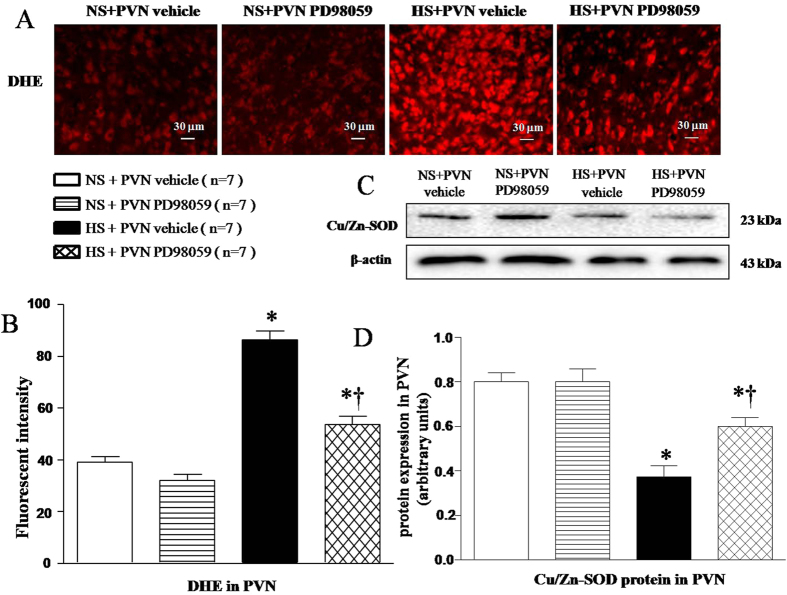
Effects of PVN infusion of PD98059 on PVN level of superoxide within the PVN in salt-induced hypertensive rats. (**A**) A representative immunofluorescence image of fluorescent-labeled dihydroethidium (DHE). (**B**) Densitometric analysis of DHE staining. (**C**) A representative immunoblot image of Cu/Zn-SOD. (**D**) Densitometric analysis of protein expression of Cu/Zn-SOD. High-salt diet (HS) rats showed stronger fluorescence intensity labeled with DHE and lower Cu/Zn-SOD proteins compared with normal-salt (NS) rats. PVN infusion PD98059 significantly decreased immunofluorescent intensity of DHE and increased Cu/Zn-SOD protein expression (*P* < 0.05, n = 7). Values are mean ± SE. *P < 0.05 vs control (NS + PVN aCSF or NS + PVN PD98059); ^†^*P* < 0.05, HS + PVN PD98059 vs HS + PVN aCSF.

Immunofluorescence stainning was performed to measure fluorescence-labelled dihydroethidium (DHE) activity to evaluate superoxide in PVN. Cu/Zn-SOD protein expression was measured by Western blot. Results shown in Fig. 4 found that HS rats had more reactive oxygen species (ROS) (Fig. 4A and B, *P* < 0.05) and less Cu/Zn-SOD protein expression (Fig. 4C and D, *P* < 0.05) in the PVN compared with NS rats. With 6-weekPVN infusion of PD-98059, these changes in HS rats were attenuated (Fig. 4, *P* < 0.05).

### Effects of PVN infusions of PD-98059 on NOX2 expression in the PVN of salt-induced hypertensive rats

**Figure 5:**
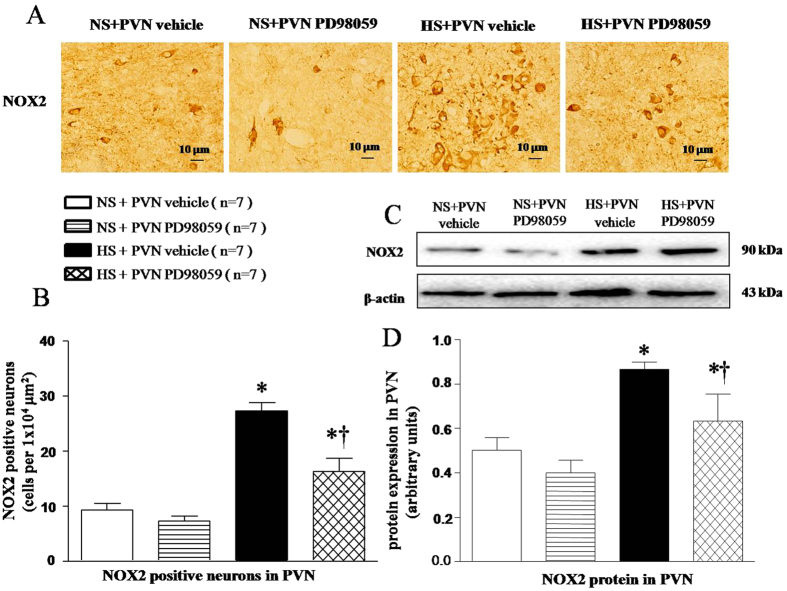
Effects of PVN infusion of PD98059 on PVN NOX2, a NAD (P) H oxidase subunit, in salt-induced hypertensive rats. (**A**) A representative immunofluorescence image of NOX2. (**B**) Densitometric analysis of NOX2. (**C**) A representative immunoblot image of NOX2. (**D**) Densitometric analysis of protein expression of NOX2. High-salt diet (HS) rats had higher levels of NOX2 compared with normal-salt (NS) rats (*P* < 0.05, n = 7). PVN infusion of PD98059 significantly attenuated NOX2 level within the PVN of HS rats (*P* < 0.05, n = 7). Values are mean ± SE. *P < 0.05 vs control (NS + PVN aCSF or NS + PVN PD98059); ^†^*P* < 0.05, HS + PVN PD98059 vs HS + PVN aCSF.

Immunofluorescence and Western blot results found that HS rats had higher level of NOX2 positive neurons (Fig. 5A and B, *P* < 0.05) and NOX2 protein expression (Fig. 5C and D, *P* < 0.05) in the PVN. With 6-week PVN infusion of PD-98059, NOX2 expression was attenuated in HS rats (Fig. 5, *P* < 0.05).

### Effects of PVN infusions of PD-98059 on NOX4 expression in the PVN of salt-induced hypertensive rats

**Figure 6:**
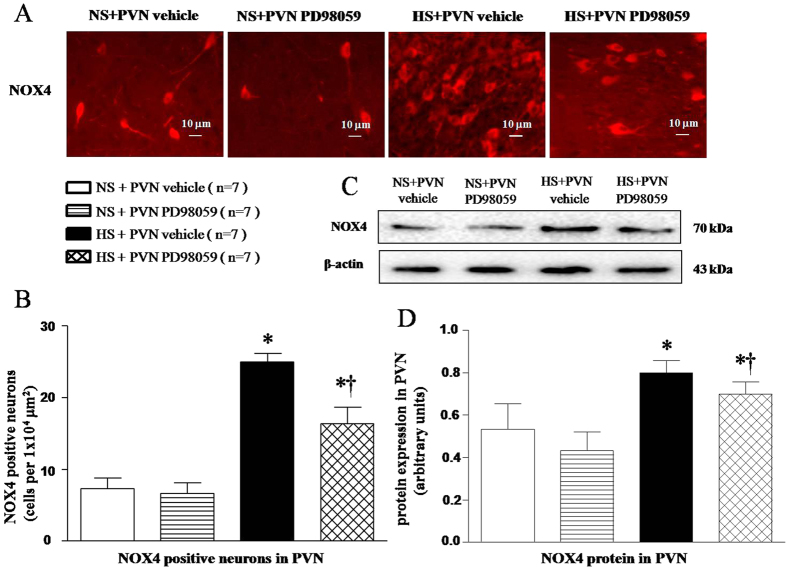
Effects of PVN infusion of PD98059 on PVN NOX4, a NAD (P) H oxidase subunit in salt-induced hypertensive rats. (**A**) A representative immunofluorescence image of NOX4. (**B**) Densitometric analysis of NOX4. (**C**) A representative immunoblot image of NOX4. (**D**) Densitometric analysis of protein expression of NOX4. High-salt diet (HS) rats had higher levels of NOX4 compared with normal-salt (NS) rats (*P* < 0.05, n = 7). PVN infusion of PD98059 significantly attenuated NOX4 level within the PVN of HS rats (*P* < 0.05, n = 7). Values are mean ± SE. *P < 0.05 vs control (NS + PVN aCSF or NS + PVN PD98059); ^†^*P* < 0.05, HS + PVN PD98059 vs HS + PVN aCSF.

Immunofluorescence and Western blot results found that HS rats had higher level of NOX4 positive neurons (Fig. 6A and B, *P* < 0.05) and NOX4 protein expression in the PVN (Fig. 6C and D, *P* < 0.05). With 6-week PVN infusion of PD-98059, NOX4 expression was attenuated in HS rats (Fig. 6, *P* < 0.05).

### Effect of PVN infusions of PD-98059 on the expression of tyrosine hydroxylase and GAD67 in the PVN of salt-induced hypertensive rats

**Figure 7:**
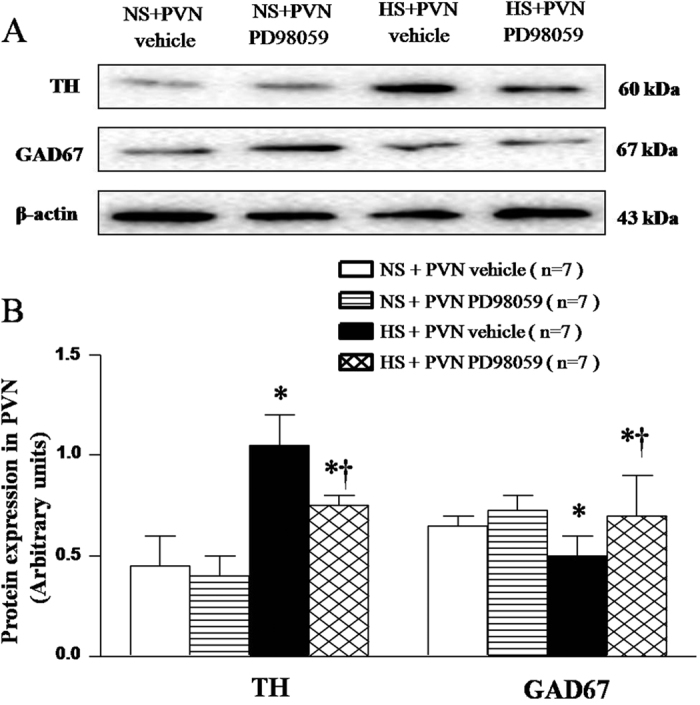
Effects of PVN infusion of PD98059 on the PVN TH and GAD67 in salt-induced hypertensive rats. (**A**) A representative immunoblot. (**B**) Densitometric analysis of protein expression of TH and GAD67. High-salt diet (HS) rats had higher levels of TH and lower level of GAD67 compared with normal-salt (NS) rats (*P* < 0.05, n = 7). PVN infusion of PD98059 significantly attenuated expression of TH, and augmented expression of GAD67 in PVN of HS rats compared with control (*P* < 0.05, n = 7). Values are mean ± SE. *P < 0.05 vs control (NS + PVN aCSF or NS + PVN PD98059); ^†^*P* < 0.05, HS + PVN PD98059 vs HS + PVN aCSF.

PVN Tyrosine hydroxylase (TH) and GAD67 expressions were measured by Western blot. As shown in Fig. 7, HS rats had higher protein expression of TH and lower GAD67 in the PVN compared with the NS rats. With 6-week PVN infusion of PD-98059 reduced the expression of TH, increased the expression of GAD67 in the PVN of HS rats (Fig. 7, *P* < 0.05).

### Effects of PVN infusions of p44/42 MAPK inhibitor PD-98059 on the neurotransmitters in the PVN

**Figure 8:**
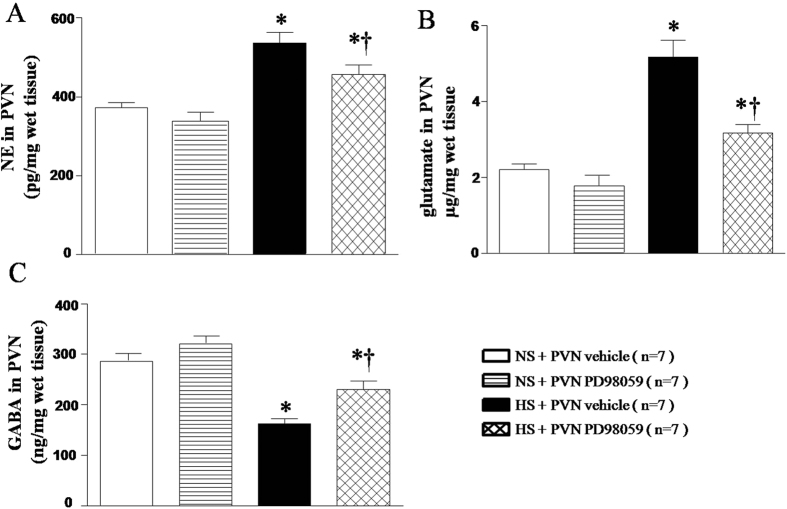
Effects of PVN infusion of PD98059 on the PVN norepinephrine, glutamate and GABA in salt-induced hypertensive rats. (**A**) norepinephrine (NE). (**B**) glutamate. (**C**) GABA. Results of HPLC showed that High-salt diet (HS) rats had higher levels of NE and glutamate and lower level of GABA compared with normal-salt (NS) rats (*P* < 0.05, n = 7). PVN infusion of PD98059 significantly attenuated level of NE and glutamate, and augmented level of GABA in PVN of HS rats compared with control (*P* < 0.05, n = 7). Values are mean ± SE. *P < 0.05 vs control (NS + PVN aCSF or NS + PVN PD98059); ^†^*P* < 0.05 HS + PVN PD98059 vs HS + PVN aCSF.

Neurotransmitters were measured by HPLC. As shown in Fig. 8, HS rats had higher levels of NE (Fig. 8A, *P* < *0.05*) and glutamate (Fig. 8B, *P* < *0.05*), and lower level of GABA (Fig. 8C, *P* < *0.05*) in the PVN compared with the NS rats. With 6-week PVN infusion of PD-98059 significantly attenuated the changes of these neurotransmitters in the PVN of HS rats (Fig. 8, *P* < 0.05).

### Effects of PVN infusion of PD-98059 on the expression of total p44/42 and ph-p44/42 in the PVN of salt-induced hypertensive rats

**Figure 9:**
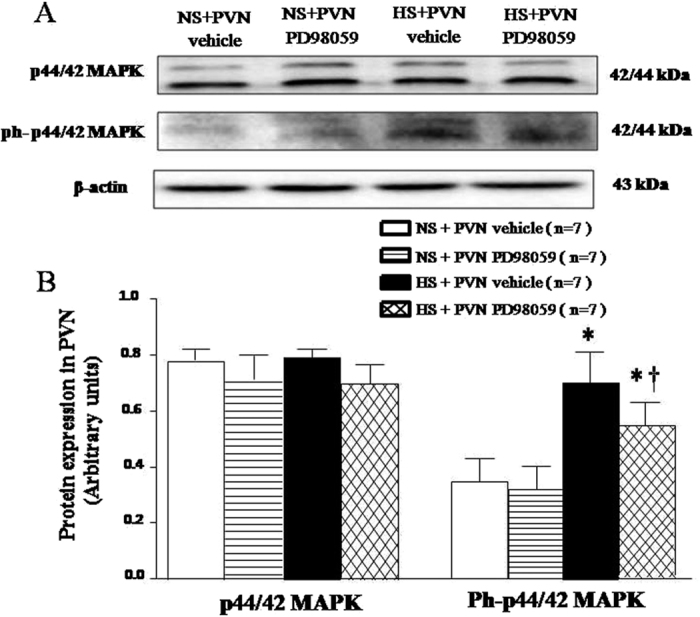
Effects of PVN infusion of PD98059 on total p44/42 and ph-p44/42 proteins expression in salt-induced hypertensive rats. (**A**) Representative immunoblots. (**B**) Densitometric analysis of protein expression of p44/42. High-salt diet (HS) rats had higher level of ph-p44/42 proteins expression in the PVN compare with normal-salt (NS) rats (*P* < 0.05, n = 7). PVN infusion of PD98059 significantly decreased the PVN level of ph-p44/42 proteins expression in HS rats compared with control (*P* < 0.05, n = 7). Values are mean ± SE. *P < 0.05 vs control (NS + PVN aCSF or NS + PVN PD98059); ^†^*P* < 0.05, HS + PVN PD98059 vs HS + PVN aCSF.

Total p44/42 and ph-p44/42 expression were measured by Western blot. As shown in Fig. 9, HS rats had no statistical difference of total p44/42 (Fig. 9A and B, *P* > 0.05) and had higher level of ph-p44/42 (Fig. 9A and C, *P* < 0.05) in the PVN. With 6-week PVN infusion of PD-98059 significantly reduced the ph-p44/42 in the PVN of HS rats (Fig. 9, *P* < 0.05).

## Discussion

The novel findings of the present study are: (i) The p44/42 MAPK (ERK1/2) pathway in the PVN was activated in high salt-induced hypertension; (ii) PVN infusion of PD98059 inhibited p44/42 MAPK pathway and reduced neuronal activity; (iii) PVN infusion of PD98059 attenuated hypertensive responses through reducing ROS and restoring the balance of neurotransmitters in the PVN of hypertensive rats.

Previous studies have found that intracerebroventricular (ICV) administration of PD98059 and UO126, two selective p44/42 MAPK inhibitors, induced significant decreases of MAP and repressed sympathetic excitation in heart failure (HF) rats^20^. Treatment with the p44/42 MAPK inhibitors proved that p44/42 MAPK (ERK1/2) mediated ANG II induced effects on sympathetic nerve activity and hemodynamics in HF rats. All the above data supported a critical role of the p44/42 MAPK signaling cascade in the maintenance of renal sympathetic excitation. Fra-LI activity, a marker of neuronal excitation that has been used to identify chronically activated neurons in PVN^34^,^35^. Consistent with their effects on peripheral sympathetic nerve activity, in this study, we found the p44/42 inhibitors also reduced neuronal excitation, as indicated by Fra-LI activity, in the PVN of hypertensive rats. The results fully demonstrated the PVN infusion of p44/42 MAPK inhibitor PD98059 induced a reduction in Fra-LI positive neurons in the PVN and attenuated hypertensive response and sympathetic activity in high salt-induced hypertensive rats.

Accumulating evidences support that salt-induced hypertension is closely related to the ROS overproduction in the PVN, which plays an important role in modulating the progression of hypertension and sympathetic nerve activity^4^,^17^. NAD(P)H subunits (especially NOX2 and NOX4) in PVN are the main sources of ROS. In the present study, we found that expressions of NOX2 and NOX4 in PVN of HS group were significantly increased compared with that in NS rats. PVN infusion p44/42 MAPK inhibitor PD98059 decreased the levels of NOX2 and NOX4 in PVN. Superoxide generation was determined by fluorescent-labelled dihydroethidium (DHE; Molecular Probes) staining was also significantly increased in comparison with that in NS rats. Based on the above results we speculate that p44/42 MAPK may increase sympathoexcitation and blood pressure by triggering the overproduction of ROS and contributes to the development of hypertension.

The prototypic members of the MAPK family, ERK1 (p44 MAPK) and ERK2 (p42 MAPK) are activated in response to some neurotransmitters. Studies over the last several decades have established that the balance between the neurotransmitters plays an important role in the rise of blood pressure^17^. It is well known that the PVN is a principal cardiovascular regulatory center and contains excitatory and inhibitory neurotransmitters^36^. Increasing evidences demonstrate that hypertension is found to be associated with increased levels of excitatory neurotransmitters and decreased levels of inhibitory GABAergic system in the PVN^18^. In this study, we demonstrated that chronic PVN infusion of p44/42 MAPK inhibitor PD98059 decreased glutamate, NE, TH and increased GABA and GAD67 (a marker to recognize GABAergic neurons) expressions in the PVN of HS rats. These results provide sufficient evidences that PVN infusion p44/42 MAPK inhibitor PD98059 may restore the balance between excitatory and inhibitory neurotransmitters in the PVN, leading to reduced blood pressure and sympathetic nerve activity in salt-induced hypertensive rats.

**Figure 10:**
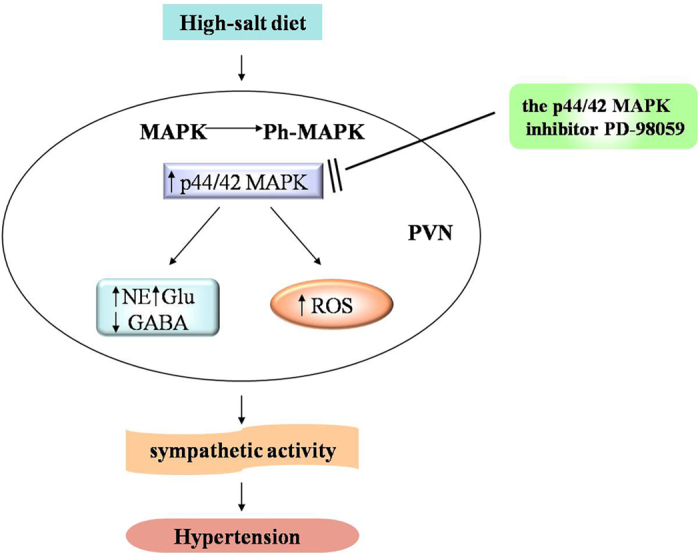
The schematic of the hypothesis showing the mechanism by which high-salt diet induces hypertension. The p44/42 MAPK (ERK1/2) pathway in the PVN was activated in high salt-induced hypertension, which results in the imbalance of neurotransmitters and overloads of ROS in the PVN. These changes and their interactions cause sympathoexcitation and eventually accelerate progression of hypertension. Treatment with PD98059 to block p44/42 MAPK pathway could reverse the pathophysiological process of hypertension through attenuating oxidative stress, and restoring the balance of neurotransmitters in PVN.

In summary, this study identified an intracellular p44/42 MAPK pathway in the PVN was activated in high-salt induced hypertension. Central blockade of PVN p44/42 MAPK pathway has also been shown to ameliorate sympathetic activation and hypertension. More importantly, PVN blockade of p44/42 MAPK pathway attenuates hypertension possibly by decreasing ROS and restoring the balance of neurotransmitters (Fig. 10), However, further mechanism of interactions between neurotransmitters and ROS within the PVN by p44/42 MAPK is warranted.

## Additional Information

**How to cite this article:** Gao, H.-L. *et al*. PVN blockade of p44/42 MAPK pathway attenuates salt-induced hypertension through modulating neurotransmitters and attenuating oxidative stress. *Sci. Rep.* **7**, 43038; doi: 10.1038/srep43038 (2017).

**Publisher's note:** Springer Nature remains neutral with regard to jurisdictional claims in published maps and institutional affiliations.
